# How vaccinia virus has evolved to subvert the host immune response

**DOI:** 10.1016/j.jsb.2011.03.010

**Published:** 2011-08

**Authors:** Mohammad W. Bahar, Stephen C. Graham, Ron A.-J. Chen, Samantha Cooray, Geoffrey L. Smith, David I. Stuart, Jonathan M. Grimes

**Affiliations:** a Division of Structural Biology and Oxford Protein Production Facility, Wellcome Trust Centre for Human Genetics, University of Oxford, Roosevelt Drive, Oxford OX3 7BN, United Kingdom; b Department of Virology, Faculty of Medicine, Imperial College London, St. Mary’s Campus, Norfolk Place, London W2 1PG, United Kingdom; c Science Division, Diamond Light Source Ltd., Diamond House, Harwell Science and Innovation Campus, Didcot, Oxfordshire 0X11 0DE, United Kingdom

**Keywords:** Bcl-2, B-cell lymphoma-2, CPXV, Cowpox virus, dsDNA, double-stranded DNA, ECTV, ectromelia virus, GAGs, glycosaminoglycans, GPCRs, G-protein coupled receptors, IFN, interferon, IG, immunoglobulin, PDB, protein data bank, RPXV, rabbitpox virus, r.m.s.d., root mean square deviation, SPINE, Structural Proteomics In Europe, TLR, Toll-like receptors, TNF, tumour necrosis factor, TNFR, tumour necrosis factor receptor, VACV, vaccinia virus, vCCI, viral CC-chemokine inhibitor, eIF2α, eukaryotic translation initiation factor 2 alpha, TRAF6, TNF-receptor-associated factor 6, IRAKs, IL-1 receptor associated kinases, IKK, IκB kinase, Structural virology, Innate immunity, Cell signalling, X-ray crystallography, Surface receptors

## Abstract

Viruses are obligate intracellular parasites and are some of the most rapidly evolving and diverse pathogens encountered by the host immune system. Large complicated viruses, such as poxviruses, have evolved a plethora of proteins to disrupt host immune signalling in their battle against immune surveillance. Recent X-ray crystallographic analysis of these viral immunomodulators has helped form an emerging picture of the molecular details of virus-host interactions. In this review we consider some of these immune evasion strategies as they apply to poxviruses, from a structural perspective, with specific examples from the European SPINE2-Complexes initiative. Structures of poxvirus immunomodulators reveal the capacity of viruses to mimic and compete against the host immune system, using a diverse range of structural folds that are unique or acquired from their hosts with both enhanced and unexpectedly divergent functions.

## 1 Introduction

*Vaccinia virus* (VACV), the smallpox vaccine, is the prototype member of the *Orthopoxvirus* genus of the *Poxviridae*: a family of large, complex dsDNA viruses that replicate in the cytoplasm of host cells and form virions with a unique morphology (Condit et al., 2006; Moss, 2007). The VACV genome reflects the complexity of poxviruses in both gene composition and structure. Its linear dsDNA genome ranges from 185–200 kbp in size, with a capacity to encode around 200 distinct proteins (Lefkowitz et al., 2006; Moss, 2007). The highly conserved central portion of most poxvirus genomes contains essential genes involved in key functions such as transcription, DNA replication and virion assembly. In contrast, genes that cluster at the ends of the genome are usually species- or host-specific and encode virulence factors that modulate the host immune system (Gubser et al., 2004; Jackson et al., 2005). Analysis of poxvirus genomes has shed new light on poxvirus phylogeny and evolution (Lefkowitz et al., 2006) showing that poxvirus proteins are generally more similar to eukaryotic proteins than bacterial, suggesting that gene acquisition by horizontal gene transfer from their eukaryotic hosts has been a slow but ongoing process that has contributed to the evolution of poxviruses (Esposito et al., 2006; Lefkowitz et al., 2006). Many of these host-derived genes facilitate poxvirus immune evasion. VACV immunomodulators function both outside and inside infected host cells. Proteins that are secreted from infected cells are directed toward binding and disrupting the function of complement, interferons (IFNs), cytokines and chemokines (Alcami, 2003; Alcami and Koszinowski, 2000; McFadden and Murphy, 2000; Perdiguero and Esteban, 2009), as well as semaphorin signalling (Seet et al., 2003). Conversely, intracellular immunomodulators modulate apoptosis, the antiviral effects of IFNs, innate immune signalling and host gene transcription (Haga and Bowie, 2005; Perdiguero and Esteban, 2009; Seet et al., 2003; Taylor and Barry, 2006).

**Fig.1:**
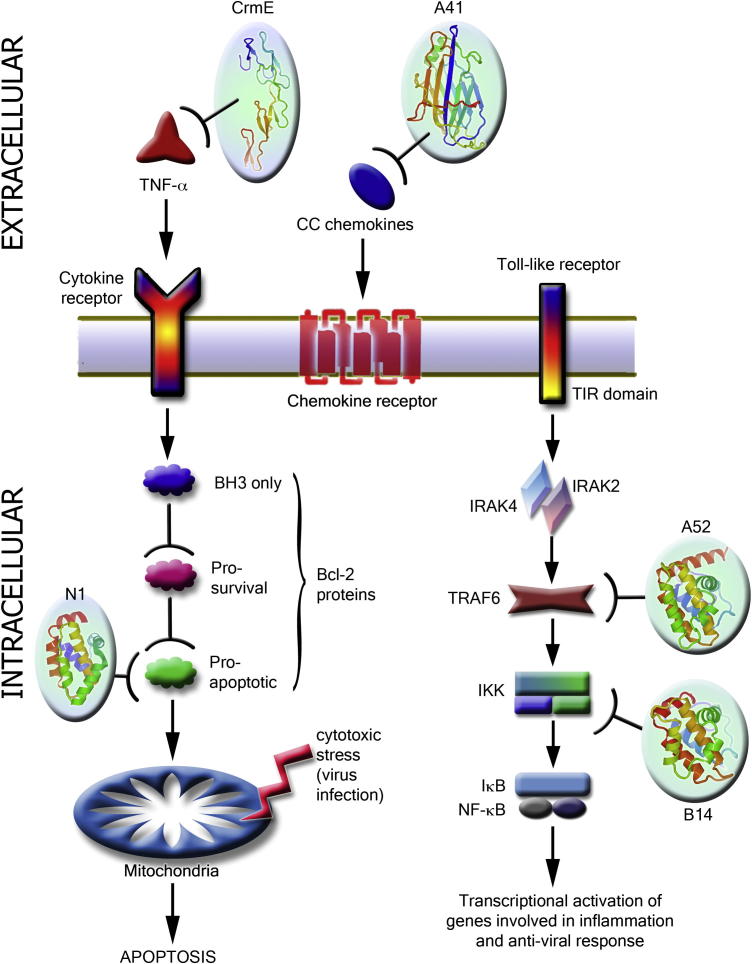
Schematic cartoon highlighting some of the anti-viral immune signalling pathways targeted by VACV. Structures of VACV proteins solved by work funded in part by SPINE2-Complexes are shown as cartoons, coloured from blue (N terminus) to red (C terminus), along with the points where they interfere with signalling.

In this review we assess the current structural knowledge on poxvirus immunomodulators (summarised in Table 1), focussing on what we have learnt from the five specific examples of extra- and intracellular immune modulators of VACV that have been solved as part of SPINE2-Complexes (shown in their functional context in Fig. 1). We highlight how VACV has evolved to use two broad classes of immunomodulators: those acquired from the host and those that appear to have no relationship to known host proteins. This work emphasises how poxviruses are able to acquire and replicate a number of structural scaffolds to carry out related but distinct immunomodulatory functions and underscores the observation that apparently unrelated sequences have often diverged from host acquired genes whilst conserving structure.

## 2 Extracellular immune evasion

Two structures of secreted VACV proteins that inhibit cytokines and chemokines were determined as part of the SPINE2-complexes activity and a number of structures have been determined by others, which are summarised first (Table 1). The crystal structure of the complex between ectromelia virus (ECTV) interleukin (IL)-18 binding protein and human IL-18 (Krumm et al., 2008) reveals that the viral protein has a canonical immunoglobulin (IG)-like fold and functions by blocking a putative receptor binding site on IL-18 (Krumm et al., 2008). The ECTV IFN-γ binding protein (IFN-γBP) complexed with IFN-γ also reveals a conservation of structure with the extracellular domain of the host IFN-γ receptor (Nuara et al., 2008). Furthermore, in this complex it was shown that ECTV IFN-γBP forms secreted tetramers that sequester two dimers of host IFN-γ. Oligomerisation of ECTV IFN-γBP is achieved through a helix-turn-helix motif that is similar in structure to the transcription factor TFIIA, demonstrating poxvirus acquisition of structural folds that have been adapted for additional functions by immunomodulators (Nuara et al., 2008). Lastly, recent structures of the VACV secreted protein A39 have provided mechanistic information about viral inhibition of semaphorins, a family of conserved signalling molecules that play crucial roles in the development of the nervous system and in immune regulation, through interactions at the cell surface with their cognate plexin receptors (Suzuki et al., 2008). VACV A39 is a secreted poxviral homolog of sema7A, and the crystal structure of the A39-PlexinC1 complex confirms that viral semaphorins share a conserved binding mode – adapted for higher affinity – to host semaphorin-plexin interactions (Liu et al., 2010, and see Bowden et al. in this issue). In each of these examples the relationship between the virus protein and its cellular counterpart(s) was deduced from comparison of primary amino acid sequence and prompted specific experiments to test function. However, in some other examples below, no such primary sequence similarity was evident and protein structure provided inference about possible function.

### 2.1 Poxvirus inhibition of TNFα – CrmE and Tanapoxvirus protein 2

**Fig.2:**
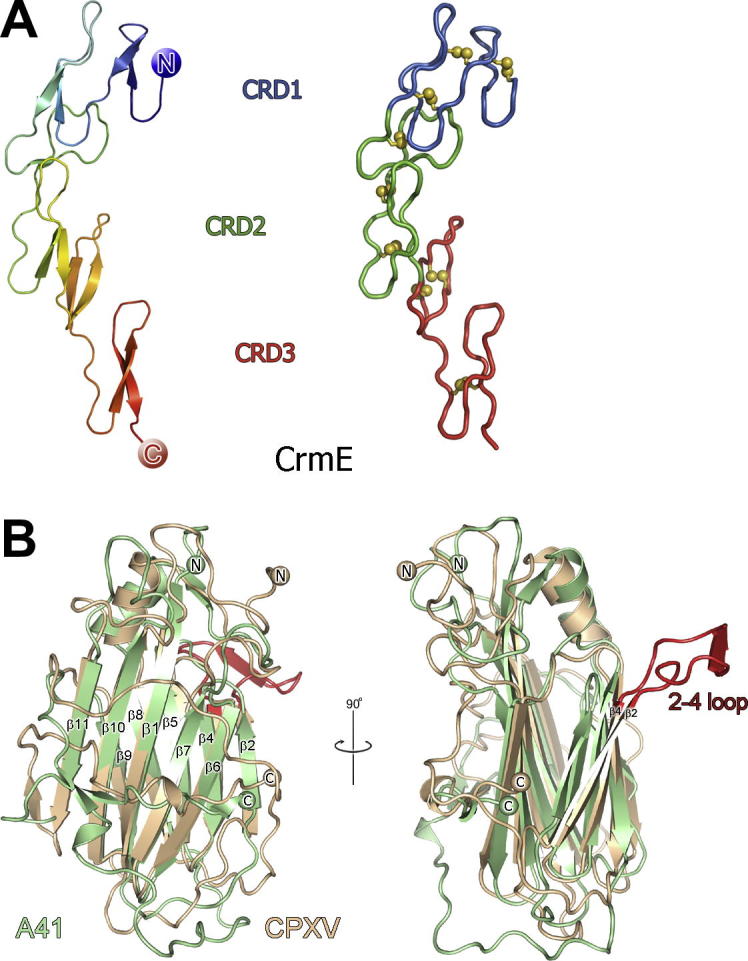
Extracellular VACV immunomodulators CrmE and A41 inhibit TNFα and CC chemokines respectively. (A) The structure of CrmE. In the left panel CrmE is shown colour ramped from blue (N terminus) to red (C terminus), and in the right panel the domain structure of CrmE is shown in a ribbon representation with CRDs 1–3 coloured blue, green and red, respectively. (B) The structure of A41 (pale green) is shown superposed onto the vCCI of cowpox virus (CPXV, wheat) with conserved β strands forming the β sheets labelled. The view in the right panel is rotated 90° with respect to the left panel and the ‘2–4 loop’ present in vCCI’s is highlighted red and labelled.

Tumour necrosis factor α (TNFα) is the prototypic member of a superfamily of potent pro-inflammatory cytokines that can induce an anti-viral state and promote apoptosis in virus-infected cells (Aggarwal, 2003; Locksley et al., 2001). Poxviruses have countered the selection pressure of the TNF superfamily by evolving proteins that disrupt TNFα-induced apoptosis (Alcami, 2003; Taylor and Barry, 2006). Two distinct classes of secreted poxvirus TNFα-binding proteins have now been characterised structurally (Table 1): those that share sequence similarity to the extracellular domain of cognate host TNF receptors (TNFRs) (Cunnion, 1999; Saraiva and Alcami, 2001) and, more recently, a separate protein that resembles the mammalian MHC class I heavy chain (Brunetti et al., 2003). The structure of VACV cytokine response modifier E (CrmE), solved as part of SPINE2-Complexes, was the first crystal structure of a virus-encoded TNFR (Graham et al., 2007). CrmE shares sequence identity (30%) with the human TNFR superfamily proteins 1A (TNFRSF1A), and the structure of CrmE (Figs. 1 and 2) adopts the canonical fold of the TNFR family of proteins, comprising three cysteine-rich domains (CRDs 1–3). Each CRD contains three intra-domain disulphide bonds (Fig. 2). The structure of CrmE has shown that only one of the two ligand-binding loops present in human TNF receptors are conserved in this viral counterpart. This has provided a structural basis for understanding the higher affinities of poxvirus TNFRs for the cytokine TNFα over other cytokines such as lymphotoxin-α (Graham et al., 2007), which is supported by the recent structure of TNFR2 in complex with TNFα (Mukai et al., 2010). In contrast to VACV CrmE, the TPXV protein 2 binds TNFα to inhibit host antiviral responses yet does not share sequence similarity to host TNFRs (Brunetti et al., 2003). The recent structure of a TPXV 2-TNFα complex has revealed that the TPXV protein 2 adopts an MHC class I structural fold but lacks a peptide binding groove for antigen presentation (Yang et al., 2009). The high-affinity binding between TPXV protein 2 and TNFα (Brunetti et al., 2003) is explained structurally by similarities in the TNF binding sites on TPXV protein 2 and host TNFRs despite different structural folds. The structure of both CrmE and TPXV 2 demonstrate modification of host structural folds to confer adapted specificities for the host TNFα ligand.

### 2.2 Poxvirus inhibition of chemokine signalling – A41 and other vCCIs

Chemokines are secreted proteins (classified into C, CC, CXC and CX3C subfamilies) that bind cell surface GAGs to form concentration gradients on vascular endothelial cell surfaces that direct the migration of leukocytes into areas of infection and inflammation (Charo and Ransohoff, 2006; Rot and von Andrian, 2004; Zlotnik and Yoshie, 2000). Several poxviruses encode a secreted viral CC chemokine inhibitor (vCCI) that prevents the interaction of chemokines with their host receptors on leukocytes.

Structures of vCCI proteins from cowpox virus (CPXV) (Carfi et al., 1999), rabbitpox virus (RPXV) (Zhang et al., 2006) and ECTV (Arnold and Fremont, 2006) (Table 1) reveal a distinct β sandwich topology with no obvious relationship to host chemokine receptors, which are seven-transmembrane G-protein coupled receptors (GPCRs). The vCCI β sandwich exposes two β sheets; sheet I is flat and electrostatically bland, whereas the second face (sheet II) projects an elaborate conserved loop that forms a negatively-charged surface. The acidic surface residues of sheet II are highly conserved in vCCI proteins and the structure of the complex between RPXV vCCI and human CCL4 has shown that these residues are the chemokine-binding surface for vCCI proteins (Zhang et al., 2006). Furthermore, structure-based mutational analysis of the ECTV vCCI protein highlighted residues involved in high affinity interactions between vCCIs and their CC chemokine partners (Arnold and Fremont, 2006).

The VACV A41 protein (studied as part of SPINE2-complexes) is secreted from infected cells, affects the host response to infection and has limited sequence similarity to CPXV and ECTV vCCIs (∼20% identity) (Clark et al., 2006; Ng et al., 2001). The 1.9 Å crystal structure of VACV A41 shows considerable similarity to vCCI proteins from other poxviruses (Bahar et al., 2008) (Figs. 1 and 2). The core β sandwich topology is conserved (2.4 Å r.m.s.d for 159 matching Cα atoms between A41 and CPXV), as are the disulphide-forming cysteine residues present in A41 and vCCIs. The most notable deviation between the structures of vCCIs and A41 lies in the topology of β sheet II (Fig. 2). CPXV vCCI contains an extended and highly acidic loop (the 2–4 loop) between β strands 2–4 that contributes significant negative charge to β sheet II (Carfi et al., 1999). This loop is conserved in both the RPXV (Zhang et al., 2006) and ECTV (Arnold and Fremont, 2006) vCCI structures but is absent in A41. In the solution structure of the complex between RPXV and the CC chemokine CCL4 the 2–4 loop forms a binding site for the chemokine through electrostatic interactions (Zhang et al., 2006).

Functional results demonstrate that A41 binds a small subset of CC chemokines (CCL21, 25, 26 and 28) with an affinity that is 1–3 orders of magnitude lower (nM) than that of vCCI proteins for a wide range of CC chemokines (pM) (Bahar et al., 2008; Ruiz-Arguello et al., 2008). Consequently, A41 does not disrupt the high affinity interactions of chemokines with their cellular receptors and is unable to inhibit leukocyte chemotaxis in response to these chemokines (Bahar et al., 2008). High concentrations of GAGs such as heparin and dextran sulphate disrupt the A41-chemokine interaction suggesting that A41 functions by binding chemokines on their GAG binding site rather than their receptor binding site. The absence of the 2–4 loop in the structure of A41 is notable, since it may help explain the selectivity of A41 for only a subset of chemokines, and modelling of A41-chemokine binding suggests that the interaction is otherwise similar to that seen between vCCIs and their chemokine partners (Bahar et al., 2008). Structures of A41 and other poxvirus vCCIs have shown that poxviruses have modified a single core structural fold to comprehensively block separate aspects of chemokine-induced inflammatory responses; high affinity (pM) chemokine interactions with their receptors are blocked by vCCIs and lower affinity (nM) chemokine–GAG interactions are inhibited by A41 disrupting chemokine gradients.

## 3 Intracellular immune evasion

In contrast to extracellular immunomodulators, poxvirus inhibition of intracellular immune signalling targets not only the downstream signalling events from cytokines such as TNFα and IFNγ, but more prominently exerts control over apoptosis; the programmed form of cell death that eradicates damaged or pathogen-infected cells (Everett and McFadden, 2002; Taylor and Barry, 2006). Three structures of intracellular signalling modulators have been determined in the course of SPINE2-complexes and additional structures have been determined by others (Table 1), and these are considered first.

Downstream IFN-signalling pathways activate the transcription factor STAT1 (signal transducer and activator of transcription 1) (Levy and Darnell, 2002), which induces the expression of important antiviral genes (Krause and Pestka, 2007). VACV VH1 protein is a dual specificity phosphatase that inhibits STAT1 by dephosphorylation (Mann et al., 2008; Najarro et al., 2001). The 1.3 Å crystal structure of VACV VH1 reveals a homodimer assembly with a prominent N-terminal helix domain swap that exposes two phosphatase active sites (Koksal et al., 2009). Functional work in the same study also showed that VH1 dephosphorylates STAT1 in the absence of bound DNA, suggesting that the VACV VH1 protein acts on cytoplasmic pools of STAT1 prior to nuclear translocation (Koksal et al., 2009). One of the genes induced in part by STAT1 is the dsRNA-dependent protein kinase (PKR), which detects dsRNA produced during VACV transcription and in response phosphorylates and inhibits the host protein translation factor eukaryotic translation initiation factor 2 alpha (eIF2α). This arrests synthesis of viral and host proteins in infected cells, leading to apoptosis (Garcia et al., 2006; Gil and Esteban, 2000). The VACV K3 protein is a viral mimic of the N-terminal 88 amino acids of eIF2α and circumvents PKR-induced apoptosis by binding to PKR and acting as a non-phosphorylable pseudosubstrate of PKR (Beattie et al., 1991; Carroll et al., 1993). The structure of K3 consists of a five-stranded β-barrel and mutational analysis of the structure reveals that two separate regions of K3 are responsible for binding PKR and inhibiting its phosphorylation of eIF2α (Dar and Sicheri, 2002).

Apoptosis represents an important host innate immune response to infection and aids elimination of virus-infected cells (Roulston et al., 1999). The B-cell lymphoma 2 (Bcl-2) family are small α-helical proteins that are either pro- or anti-apoptotic, regulating the release of pro-apoptotic molecules from mitochondria (Youle and Strasser, 2008). Unsurprisingly, several viruses, including adenovirus and herpesvirus, express anti-apoptotic viral Bcl-2 proteins to evade cell death (Polster et al., 2004). Recent functional and structural data has identified a family of poxviral proteins that lack sequence similarity to cellular or viral Bcl-2 proteins yet adopt a Bcl-2 fold and act as potent anti-apoptotic Bcl-2 proteins (Table 1). Crystallographic analysis of these poxvirus Bcl-2–like proteins has revealed details of viral inhibition of apoptosis and, for specific examples from VACV, has also revealed unexpected adaptations of Bcl-2-folds for wider intracellular signal modulation, discussed below (Table 1).

### 3.1 Poxvirus Bcl-2–like proteins that block apoptosis – N1, M11 and F1

**Fig.3:**
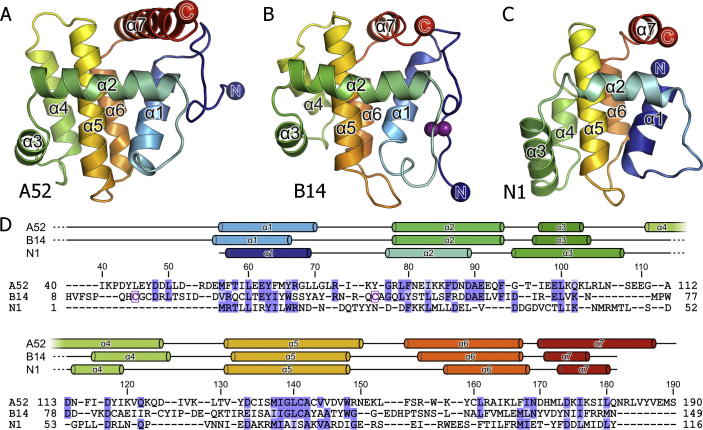
VACV proteins A52, B14 and N1 share a Bcl-2–like fold. (A) A52, (B) B14 and (C) N1 are shown as ribbons, rainbow coloured from blue (N terminus) to red (C terminus). The disulphide bond observed in B14 is shown as purple spheres. (D) Structure-based sequence alignment of the VACV Bcl-2–like proteins. Residues that are highly or moderately conserved (BLOSUM62 score) are coloured marine and light blue, respectively, and cysteine residues that form the disulphide bond observed in B14 are boxed and in purple face. The secondary structures of A52, B14 and N1 are shown above the sequences with α helices represented as cylinders. This figure is revised and updated from Graham et al., 2008.

In work performed as part of SPINE2-Complexes and by others, the VACV N1 protein, a VACV virulence factor (Bartlett et al., 2002), was shown to possess an α-helical fold very similar to cellular Bcl-2 family members, despite lacking detectable sequence similarity to Bcl-2 homology (BH) motifs (Fig. 3) (Aoyagi et al., 2007; Cooray et al., 2007). Similar to host anti-apoptotic Bcl-2 proteins, N1 contains conserved structural features including a prominent surface groove, which in cellular Bcl-2 proteins binds BH3 motifs of pro-apoptotic family members and inhibits their cell death functions (Hinds and Day, 2005; Liu et al., 2003; Petros et al., 2004). In cellular Bcl-2 proteins, access to the surface groove is modulated by a C-terminal helix that is displaced from the groove before pro-apoptotic Bcl-2 proteins can be sequestered (Hinds et al., 2003; Wilson-Annan et al., 2003). In contrast, the N1 C-terminal helix is truncated and the surface groove is constitutively open (Cooray et al., 2007). Molecular modelling of helical pro-apoptotic BH3 motifs into the N1 surface groove reveals that, unlike the predominantly hydrophobic grooves of its cellular counterparts, the N1 binding pocket displays some charged characteristics, suggesting that although the core binding sites are conserved, N1 may bind BH3 motifs selectively (Cooray et al., 2007). Functional work confirms that N1 inhibits apoptosis in VACV-infected cells, and that N1 interacts with pro-apoptotic Bcl-2 proteins such as Bid, Bad, Bak and Bax *in vivo* (Cooray et al., 2007) and *in vitro* (Aoyagi et al., 2007). In addition, the N1 protein has been reported to inhibit innate immune signalling pathways by binding to both the IKK complex and TANK binding kinase 1 (TBK1) and thereby inhibit activation of nuclear factor (NF)-κB and IRF3 (DiPerna et al., 2004).

Myxoma virus (MYXV) M11 and VACV F1 have also been identified as poxvirus proteins that inhibit apoptosis (Everett et al., 2002; Fischer et al., 2006) and recent structures have confirmed they are viral Bcl-2 family proteins. The X-ray structure of the MYXV M11 protein (Douglas et al., 2007; Kvansakul et al., 2007) reveals that, despite lacking sequence similarity to other Bcl-2 proteins, it adopts an entirely α-helical fold similar to VACV N1 and host Bcl-2 proteins. Fluorescence polarization assays have shown that M11 binds BH3 peptides of pro-apoptotic Bak with a comparable affinity to some cellular Bcl-2 proteins (Douglas et al., 2007) and the crystal structure of M11 in complex with the BH3 peptide of Bak confirms a binding mode similar to cellular Bcl-2 members; the BH3 helix of Bak binding into the hydrophobic surface groove of M11 (Kvansakul et al., 2007). The X-ray crystal structure of VACV F1, reveals a conserved core Bcl-2 fold, but also a novel domain-swapped mode of dimerisation, via an extended N-terminal region, not observed in other host or viral Bcl-2 proteins (Kvansakul et al., 2008). Previous binding data for F1 have shown that F1 binds directly to the BH3 motifs of pro-apoptotic Bcl-2 proteins with affinities ranging from 75 nM to over 1 μM (Fischer et al., 2006). Unlike VACV N1, both M11 and F1 contain an extended C-terminal hydrophobic helix that localises them to the mitochondrial membrane where they inhibit apoptosis (Everett et al., 2002; Stewart et al., 2005). Host pro-apoptotic Bcl-2 proteins such as Bak and Bax initiate apoptosis at the mitochondrial membrane (Griffiths et al., 1999; Hsu and Youle, 1998), and the presence of poxviral Bcl-2 proteins with (M11 and F1) and without (N1) membrane localising C-terminal helices suggest poxviruses have targeted the Bcl-2 pathway at both the cytosolic and mitochondrial membrane levels.

### 3.2 Poxvirus Bcl-2–like proteins that inhibit NF-κB pathway – A52, B14, N1 and K7

Nuclear factor (NF)-κB is a transcription factor complex that plays a central role in stimulating innate and adaptive immune responses to infection. Receptors for the pro-inflammatory cytokines IL-1 and TNFα activate signalling pathways leading to NF-κB activation (Hayden and Ghosh, 2008; Hayden et al., 2006), as do Toll-like receptors (TLRs), which recognise pathogen associated molecular patterns in, for example, viral (glyco)proteins and nucleic acids (Akira et al., 2006; Kawai and Akira, 2007). NF-κB activation downstream of the IL-1 receptor and TLRs requires TNF-receptor-associated factor 6 (TRAF6) and IL-1 receptor associated kinases (IRAKs), while activation downstream of the TNF receptor requires TRAF2 (Akira et al., 2006; Hayden et al., 2006). These independent downstream signalling pathways converge at the IκB kinase (IKK) complex, a key regulator of signalling to NF-κB activation (Hayden and Ghosh, 2008). The importance of the inflammatory immune response initiated by NF-κB is underscored by the fact that VACV encodes several proteins, A52, B14, N1, and K7, that inhibit aspects of the NF-κB signalling pathway (Chen et al., 2008; Harte et al., 2003; Schroder et al., 2008; DiPerna et al., 2004). Members of this group belong to a Pfam (Finn et al., 2008) protein family that also includes A46, C6 and N2. Although these proteins have sequence similarity permitting their grouping together, they do not share significant sequence similarity to other cellular or viral proteins (Bowie et al., 2000; Smith et al., 1991).

Recent results from SPINE2-Complexes and others show that VACV N1, A52, B14 and K7 are entirely α helical Bcl-2–like proteins (Graham et al., 2008; Kalverda et al., 2009). The structures of A52 and B14 both comprise seven α helices (Fig. 3), and the closest structural relative to both proteins is VACV N1 (Graham et al., 2008). Functional work also demonstrates that unlike N1 and MYXV M11, A52 and B14 do not inhibit mitochondrial apoptosis (Graham et al., 2008). Superposition of A52 and B14 structures onto MYXV M11 in complex with the BH3 peptide of Bak (Kvansakul et al., 2007) reveals the hydrophobic BH3-peptide binding grooves are occluded in both A52 and B14, but not in N1 (Fig. 3 and (Graham et al., 2008)), providing a molecular explanation for the inability of A52 and B14 to protect cells from apoptotic challenge. The absence of a surface groove is also observed for VACV K7, and consistent with this K7 does not inhibit apoptosis (Kalverda et al., 2009; Schroder et al., 2008). Functional work has also established that B14 acts at the IKK complex to inhibit signalling to NF-κB (Chen et al., 2008; Graham et al., 2008) whilst A52 blocks signalling upstream of B14 by inhibiting TLR-induced signalling through IRAK2 and TRAF6, consistent with previous observations (Bowie et al., 2000; Harte et al., 2003). Like A52 and B14, VACV K7 has a Bcl-2 fold (Kalverda et al., 2009) and, similar to A52, inhibits TLR-induced NF-κB activation (Schroder et al., 2008). However, unlike A52 or B14, K7 also forms a complex with the human DEAD-box RNA helicase DDX3 to antagonize IFN-β promoter induction and inhibit the production of pro-inflammatory cytokines (Kalverda et al., 2009). The structure of K7 in complex with a peptide of DDX3 visualised the molecular details of this interaction (Oda et al., 2009) and reveals that the DDX3 peptide (representing the N-terminal DDX3 residues 71–90) binds to a negatively charged surface on K7 and buries two critical phenylalanine residues (F84 and F85) into a hydrophobic pocket on the surface of K7. Mutation of these residues abolishes DDX3 binding to K7 and inhibits DDX3-induced signalling, suggesting that VACV K7 sequesters DDX3 by these critical motifs to inhibit IFN-β promoter induction. Collectively, the structural results for VACV A52, B14 and K7 provide further examples of poxviruses adapting host acquired structural scaffolds and diversifying their functional repertoire.

### 3.3 The evolution of Poxvirus Bcl-2–like proteins

**Fig.4:**
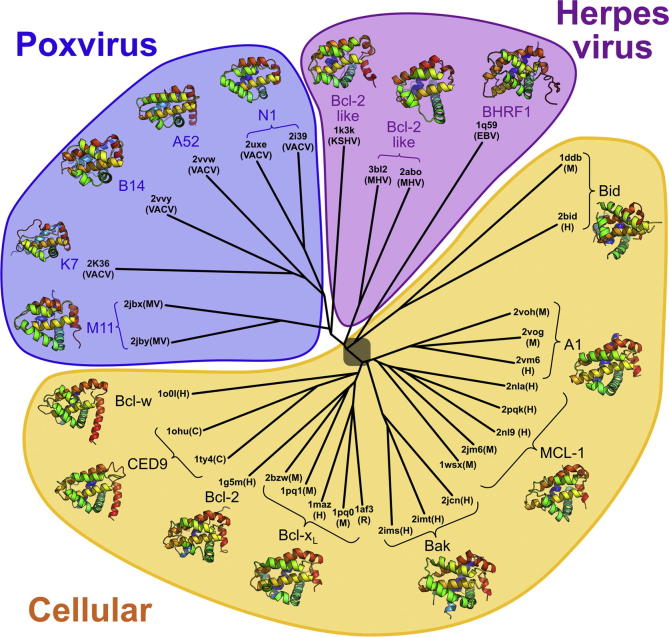
Structure-based phylogenetic analysis of virus and cellular Bcl-2–like proteins. The structures were superposed, pairwise distance matrix was constructed and tree drawn as described in (Graham et al., 2008). PDB codes for each structure used are given followed by their species of origin in parentheses [Human (H), Mouse (M), Rat (R), *C. elegans* (C), Vaccinia virus (VACV), Myxoma virus (MV), Kaposi sarcoma herpes virus (KSHV), murine gamma-herpesvirus 68 (MHV), Epstein-Barr virus (EBV)]. Although likely, it is not certain that all Bcl-2–like proteins share a common ancestor and, as such, the root of the tree is shaded. Ribbon diagrams of representative structures for each protein are rainbow coloured from blue (N terminus) to red (C terminus).

Intriguingly, the structures of N1, B14, A52, K7 and M11 suggest that Bcl-2 proteins appear to be a good structural scaffold upon which poxviruses have grafted a number of distinct immunomodulatory functions. Using a structure based approach to phylogentic analysis (Bamford et al., 2005) we have shown that viral Bcl-2 proteins are more similar to each other than to cellular Bcl-2 proteins, whilst for the cellular Bcl-2–like proteins, orthologues cluster together, residing closer to each other on the tree than to paralogue structures from the same species (Graham et al., 2008) (Fig. 4). The clustering of the Bcl-2 proteins of poxviruses and herpesviruses, which are not thought to be related, may be due to an ancient horizontal gene transfer event, since the Bcl-2 proteins from gamma-herpesvirus 68 (γHV68) and Kaposi-sarcoma herpesvirus (KSHV) diverge prior to the poxvirus proteins whilst the VACV and MYXV proteins largely cluster together. In addition, the analysis reveals that A52, B14, N1 and K7 are more closely related to each other than they are to any other Bcl-2 family member, despite playing distinct functional roles. This suggests that an ancestral poxvirus acquired a *Bcl-2* family gene from the host and that gene duplication events during poxviruses evolution gave rise to structurally-related proteins that have evolved separate and diverse functions, as observed in the case of VACV N1, B14, A52, K7 and M11 of myxoma virus. Furthermore N1, which has both anti-apoptotic and anti-signalling functions, occupies an intermediate position on the tree, branching after M11 (which is only anti-apoptotic with an open peptide binding groove) and before B14, K7 and A52 (which are only anti-signalling and possess occluded grooves).

## 4 Concluding remarks

Until recently, the structural biology of viral immune evasion proteins had progressed slowly compared to their functional and biochemical characterisation. However, a steady increase in structural data is now being achieved, and the ability of structural comparison to detect distant relationships has revealed that whilst viral immunomodulators have been placed into two broad classes: those acquired directly from the host, and those that appear to have no relation to host proteins based on sequence similarity, many members of the latter group are in fact host-protein derived. Thus, apparently unrelated sequences have often diverged from host acquired genes, whilst maintaining a conservation of 3-dimensional structure (Gewurz et al., 2001).

Poxviruses target many fundamental components of the innate immune response; namely the inflammatory response (IFNs, cytokines, chemokines) and apoptosis (death-receptor and Bcl-2 pathways). Although many of these immunomodulators have been identified based on sequence similarity to cellular products (Jackson et al., 2005; Seet et al., 2003), the results achieved through SPINE2-Complexes have shown how viral proteins with no clear resemblance to host components at the sequence level have maintained a high level of structural conservation (N1, B14 and A52) or have shown distinct folds (A41). For these proteins the structure has provided a lead for investigating the biological function, however it is also striking how the original functions of these folds have not only been maintained or enhanced but have frequently been diversified to enrich the viral arsenal against host immune assault.

It is clear from our current knowledge that structural analysis of further poxviral proteins that have unknown function and no sequence similarity to host proteins will reveal many more viral immunomodulators, further enhancing our understanding of the host immune system and the viral response, and potentially allowing us to leverage the evolutionary struggle between host and virus in order to inform the development of new therapeutics for viral and non-viral disease.

## Figures and Tables

**Table 1:** Structures of poxvirus immunomodulators present in the PDB.

	Description	References	PDB ID
*Extracellular immunomodulators*			
**A41**	Chemokine inhibitor	Bahar et al. (2008)	2VGA
CPXV vCCI	Chemokine inhibitor	Carfi et al. (1999)	1CQ3
Rabbitpox virus vCCI	Chemokine inhibitor (complex)	Zhang et al. (2006)	2FIN
ECTV vCCI	Chemokine inhibitor	Arnold and Fremont (2006)	2GRK
**CrmE**	TNF-α inhibitor	Graham et al. (2007)	2UWI
TPXV 2	MHC-like TNF-α inhibitor	Yang et al. (2009)	3IT8
IL-18 BP	Blocks IL-18 binding to IL-18R	Krumm et al. (2008)	3F62
IFN-gamma BP	Blocks IFNγ - binding to IFN-γR	Nuara et al. (2008)	3BES
A39	Semaphorin 7A mimic	Liu et al. (2010)	3NVX

*Intracellular immunomodulators*			
**N1**	Bcl-2–like inhibitor of apoptosis and toll-like receptor signalling to NF-κB	Cooray et al. (2007); Aoyagi et al. (2007)	2UXE 2I39
**B14**	Bcl-2–like inhibitor of NF-κB	Graham et al. (2008)	2VVY
**A52**	Bcl-2–like inhibitor of NF-κB	Graham et al. (2008)	2VVW
K7	Bcl-2–like inhibitor of NF-κB and IFN-β	Oda et al. (2009)	3JRV
F1	Bcl-2–like anti-apoptotic	Kvansakul et al. (2008)	2VTY
M11	Bcl-2–like anti-apoptotic	Kvansakul et al. (2007)	2JBY
K3	Inhibits PKR mediated phosphorylation of eIF2α	Dar and Sicheri (2002)	1LUZ
VH1	Dephosphorylates STAT-1, blocks expression of IFN induced genes (ISGs)	Koksal et al. (2009)	3CM3

Structures highlighted in bold were determined as part of SPINE2-Complexes.
